# Semaphorin-7A Is an Erythrocyte Receptor for *P. falciparum* Merozoite-Specific TRAP Homolog, MTRAP

**DOI:** 10.1371/journal.ppat.1003031

**Published:** 2012-11-15

**Authors:** S. Josefin Bartholdson, Leyla Y. Bustamante, Cecile Crosnier, Steven Johnson, Susan Lea, Julian C. Rayner, Gavin J. Wright

**Affiliations:** 1 Cell Surface Signalling Laboratory, Wellcome Trust Sanger Institute, Hinxton, Cambridge, United Kingdom; 2 Malaria Programme, Wellcome Trust Sanger Institute, Hinxton, Cambridge, United Kingdom; 3 Sir William Dunn School of Pathology, University of Oxford, Oxford, United Kingdom; University of Michigan, United States of America

## Abstract

The motility and invasion of *Plasmodium* parasites is believed to require a cytoplasmic actin-myosin motor associated with a cell surface ligand belonging to the TRAP (thrombospondin-related anonymous protein) family. Current models of invasion usually invoke the existence of specific receptors for the TRAP-family ligands on the surface of the host cell; however, the identities of these receptors remain largely unknown. Here, we identify the GPI-linked protein Semaphorin-7A (CD108) as an erythrocyte receptor for the *P. falciparum* merozoite-specific TRAP homolog (MTRAP) by using a systematic screening approach designed to detect extracellular protein interactions. The specificity of the interaction was demonstrated by showing that binding was saturable and by quantifying the equilibrium and kinetic biophysical binding parameters using surface plasmon resonance. We found that two MTRAP monomers interact via their tandem TSR domains with the Sema domains of a Semaphorin-7A homodimer. Known naturally-occurring polymorphisms in Semaphorin-7A did not quantitatively affect MTRAP binding nor did the presence of glycans on the receptor. Attempts to block the interaction during *in vitro* erythrocyte invasion assays using recombinant proteins and antibodies showed no significant inhibitory effect, suggesting the inaccessibility of the complex to proteinaceous blocking agents. These findings now provide important experimental evidence to support the model that parasite TRAP-family ligands interact with specific host receptors during cellular invasion.

## Author Summary

Apicomplexan parasites are one of the most significant groups of pathogens infecting humans and include *Plasmodium falciparum*, the parasite responsible for malaria. These parasites critically depend on their human host and must invade our cells to multiply; therefore, understanding this invasion process - with the eventual aim of therapeutically preventing it - has been a focus for scientific investigation. A key component of the invasion machinery is a family of proteins (the “TRAP” family) which traverse the membrane surrounding the parasite: the part remaining within the parasite connects to a molecular motor that powers invasion, whilst the surface-exposed region is thought to interact with proteins on the surface of the target host cell. One major question that remains unanswered is the identity of the host receptors for the TRAPs. In our paper, we use a method specifically designed to detect interactions that occur in the extracellular space between host and pathogen proteins to reveal a host receptor called Semaphorin-7A for the TRAP-family member used by the blood stage of the malarial parasite – a protein called MTRAP. The characterization of this host-parasite interaction may therefore lead to novel therapies based upon preventing parasite invasion.

## Introduction

*Plasmodium falciparum* is the etiological agent of the most severe form of malaria causing over one million deaths annually, primarily in African children [1]. The parasite lifecycle is complex and involves distinct stages that can recognise and invade differentiated cell types of both the human host and the mosquito vector. These stages are characterised by different invasive properties: ookinetes must cross the epithelial cells of the mosquito gut; sporozoites target both the secretory cells of the mosquito salivary glands and the hepatocytes of the human host, which they can either traverse or invade; and merozoites invade human erythrocytes. The ability of each stage to invade their target cells is an obligatory step in the lifecycle of the parasites and therefore these events have been considered attractive points for therapeutic intervention.

**Figure 1:**
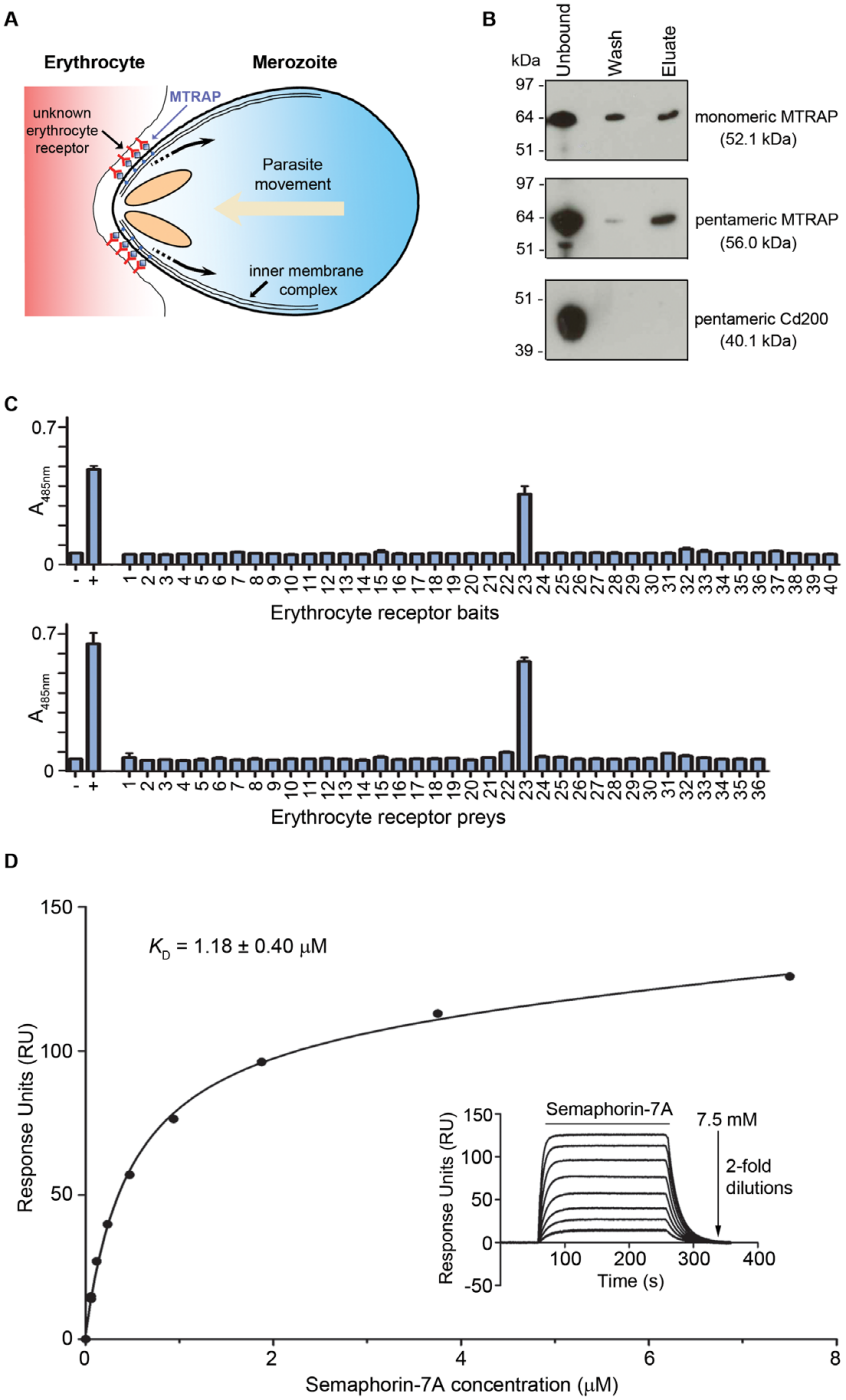
Semaphorin-7A is an erythrocyte receptor for MTRAP. (**A**) Schematic diagram showing how TRAP-like ligands are thought to play a key bridging role between the parasite and the target cell that is to be invaded. In this case, the cytoplasmic region of MTRAP interacts with the parasite actin-myosin motor that provides the power necessary for invasion. The extracellular region of MTRAP interacts with an erythrocyte receptor thereby providing the necessary traction for forwards movement of the parasite, driving host cell invasion. (**B**) Purified monomeric and pentameric MTRAP bound human erythrocytes relative to a negative control (pentameric Cd200). Unbound, wash and eluted material was resolved under reducing conditions by SDS-PAGE and detected by Western blotting using an anti-His antibody. Predicted monomer molecular weights are indicated in brackets. The pentamers are expected to split into the constituent monomers upon reduction. (**C**) Systematic screening identifies Semaphorin-7A as an MTRAP receptor. MTRAP was screened against an erythrocyte receptor protein library using the AVEXIS assay, either as a prey against 40 erythrocyte baits (top panel) or as a bait against 36 erythrocyte preys (bottom panel). A single interaction with Semaphorin-7A (protein number 23) was identified in both bait–prey orientations. Bar graphs represent means ± SD, n = 3. (**D**) MTRAP and Semaphorin-7A directly interact. Serial dilutions of purified monomeric Semaphorin-7A were injected over MTRAP immobilised on a streptavidin-coated sensor chip until equilibrium had been achieved (inset). Reference-subtracted binding data were plotted as a binding curve and the equilibrium dissociation constant was calculated using non-linear regression fitting of a simple Langmuir binding isotherm to the data. A *K*_D_ of 1.18±0.40 µM (mean ± SEM) was calculated from three independent experiments (Table S1). A representative experiment is shown.

Invasion and motility requires a single-headed class XIV myosin anchored to the inner membrane complex that unidirectionally propels short actin filaments to impart motive force [2]. The actin filaments are coupled via the glycolytic enzyme aldolase [3], [4] to parasite cell surface proteins or “invasins” belonging to the TRAP (thrombospondin-related anonymous protein) family, which in turn are thought to bind via their extracellular region to host cell surface receptors thereby coupling the actin-myosin power-stroke into forwards movement of the parasite (Figure 1 A).

Each different motile form of the parasite is distinguished by its own stage-specific cell surface TRAP-family member [5]. In *Plasmodium* species, the TRAP-family proteins include TRAP, S6 (also known as TREP), CTRP, MTRAP and TLP. TRAP and S6 are expressed on sporozoites [6], [7], [8], CTRP on ookinetes [9], MTRAP on merozoites [10] and TLP on both sporozoites and merozoites [11], [12]. Attempts to target the genes encoding these proteins have shown that most of them are essential for motility and invasion. TRAP is critical for sporozoite invasion of the salivary glands and for infection of mammalian liver as well as sporozoite gliding motility [13]; CTRP is essential for invasion of the mosquito midgut [9]; and S6 is important for sporozoite gliding motility and invasion of mosquito salivary glands [6], [8]. TLP deletion initially showed no effect indicating a redundant role for this protein [11]; however, recent studies indicate a role in sporozoite cell traversal [12], [14]. The TRAP-family can be extended to include other cell surface and secreted proteins that contain similar domains and include CSP, SPATR, TRSP, WARP and PTRAMP [5]; PTRAMP, like MTRAP, is expressed in merozoites [15]. To date, it has not been possible to genetically delete MTRAP, indicating it may be essential for parasite growth in blood stage culture [10].

Structurally, TRAP-family proteins are predicted type I cell surface proteins characterised by having one or more extracellular thrombospondin type-I repeats (TSR) domains, and/or von Willebrand factor (vWF)-like A-domain(s) and an acidic cytoplasmic tail with a sub-terminal tryptophan residue [5]. Studies of the individual domains have implicated distinct functions in motility and invasion. The cytoplasmic tail of TRAP, CTRP, TLP and MTRAP have all been shown to interact with aldolase [4], [10], [11], and the cytoplasmic tail of TRAP was shown to be essential for gliding motility and invasion of both salivary glands and hepatocytes [16]. The extracellular TSR and vWF A-domains have been implicated in host cell interaction and invasion; indeed, both the TSR and A-domain of TRAP have been shown to be essential for invasion into both mosquito salivary glands and mammalian hepatocytes [17], whereas for CTRP, only the A-domains are essential for infectivity [18]. In contrast to the cytoplasmic regions of these proteins, much less is known about the host binding partners for the extracellular regions of TRAP-like proteins and how they play a role in motility, invasion and host cell tropism. The human host extracellular molecules that have so far been identified as binding TRAP-family proteins are not restricted to particular cell types: TRAP ectodomains are known to bind sulphated glycoconjugates [7], [19] and heparin [20], [21], [22] which are both widely distributed molecules. Indeed, both biochemical and functional studies have suggested the presence of additional TRAP receptors on hepatocytes [17], [20], [23] but their identities remain unknown; very likely, this is due to the technical challenges of biochemically manipulating membrane proteins and the fact that their extracellular interactions are typified by highly transient interaction strengths [24]. The identification of the host cell surface receptor proteins for TRAP-like parasite ligands remains an important unanswered question towards a better understanding of their role in host cell recognition and invasion (Figure 1 A).

Here we report how we have used an assay that is specifically designed to circumvent the technical difficulties in identifying low affinity extracellular interactions called AVEXIS (for AVidity-based EXtracellular Interaction Screen) [25], [26] to identify Semaphorin-7A as an erythrocyte receptor for the *P. falciparum* merozoite-specific TRAP homolog protein, MTRAP. We report the biochemical characterisation of the interaction and examine its role in erythrocyte invasion.

## Results

### Semaphorin-7A is an erythrocyte receptor for *Plasmodium falciparum* MTRAP

To identify an erythrocyte receptor for *P. falciparum* MTRAP, we expressed the entire predicted extracellular region as a secreted recombinant protein in human embryonic kidney (HEK)293E cells. Given the known difficulties in expressing functional *Plasmodium* proteins [27], we codon-optimised the MTRAP gene for expression in mammalian cells, replaced the signal peptide with a high-scoring exogenous sequence from a mouse antibody, and mutated the predicted N-linked glycosylation sequons to prevent inappropriate glycan addition that might mask potential protein interaction interfaces. The ectodomain was expressed as both a monomeric and a pentameric his-tagged protein. Pentamerisation was achieved by using a peptide sequence derived from the cartilage oligomeric matrix protein (COMP) and was used to increase binding avidity so as to increase the likelihood of detecting transient binding events that are a common feature of extracellular receptor interactions. Both the monomeric and pentameric forms of MTRAP bound human erythrocytes (Figure 1 B) relative to controls, which confirmed that the proteins were biochemically active and that MTRAP binds an erythrocyte cell surface receptor. As expected, the binding of the more avid pentameric protein was more resistant to washing steps (Figure 1 B).

To determine the molecular identity of the human erythrocyte MTRAP receptor, we took a systematic approach by using the AVEXIS assay and a protein library that represents the cell surface receptor repertoire of the human erythrocyte. This approach has been successfully used to identify basigin as the erythrocyte receptor for *P. falciparum* RH5 [26]. The pentameric β-lactamase-tagged MTRAP ectodomain was screened against the library of 40 erythrocyte receptor baits used previously. A single interaction was observed (Figure 1 C, upper panel) corresponding to Semaphorin-7A (also known as CD108). The same single interaction was identified in the reciprocal bait-prey orientation (Figure 1 C, lower panel). Semaphorin-7A is a GPI-linked surface protein that is broadly expressed in several tissues [28], [29], and particularly on activated lymphocytes where it has been shown to be involved in regulating immune responses [30], [31] and neurons of both the central and peripheral nervous systems where it has documented roles in axon guidance [32]. Semaphorin-7A is the antigen for the John-Milton-Hagen blood group, although its function on erythrocytes isn't known.

To show that MTRAP and Semaphorin-7A interact directly and to quantify the biophysical parameters of the interaction, we used surface plasmon resonance (SPR). The entire ectodomain of Semaphorin-7A was expressed as a soluble recombinant protein and purified before serial dilutions were injected over MTRAP immobilised on a sensor chip. Clear saturable binding was observed (Figure 1 D) from which an equilibrium binding constant (*K*_D_) of 1.18±0.40 µM was derived. Independent kinetic parameters were in agreement with the equilibrium data (Table S1) and were within the expected range for a typical membrane-tethered receptor-ligand pair that have been shown to have physiological relevance [24], [26], [33]. Taken together, these data show that Semaphorin-7A is an erythrocyte receptor for the *P. falciparum* merozoite TRAP-family ligand, MTRAP.

### MTRAP binding to Semaphorin-7A is not influenced by glycosylation, nor does it bind glycoconjugates

**Figure 2:**
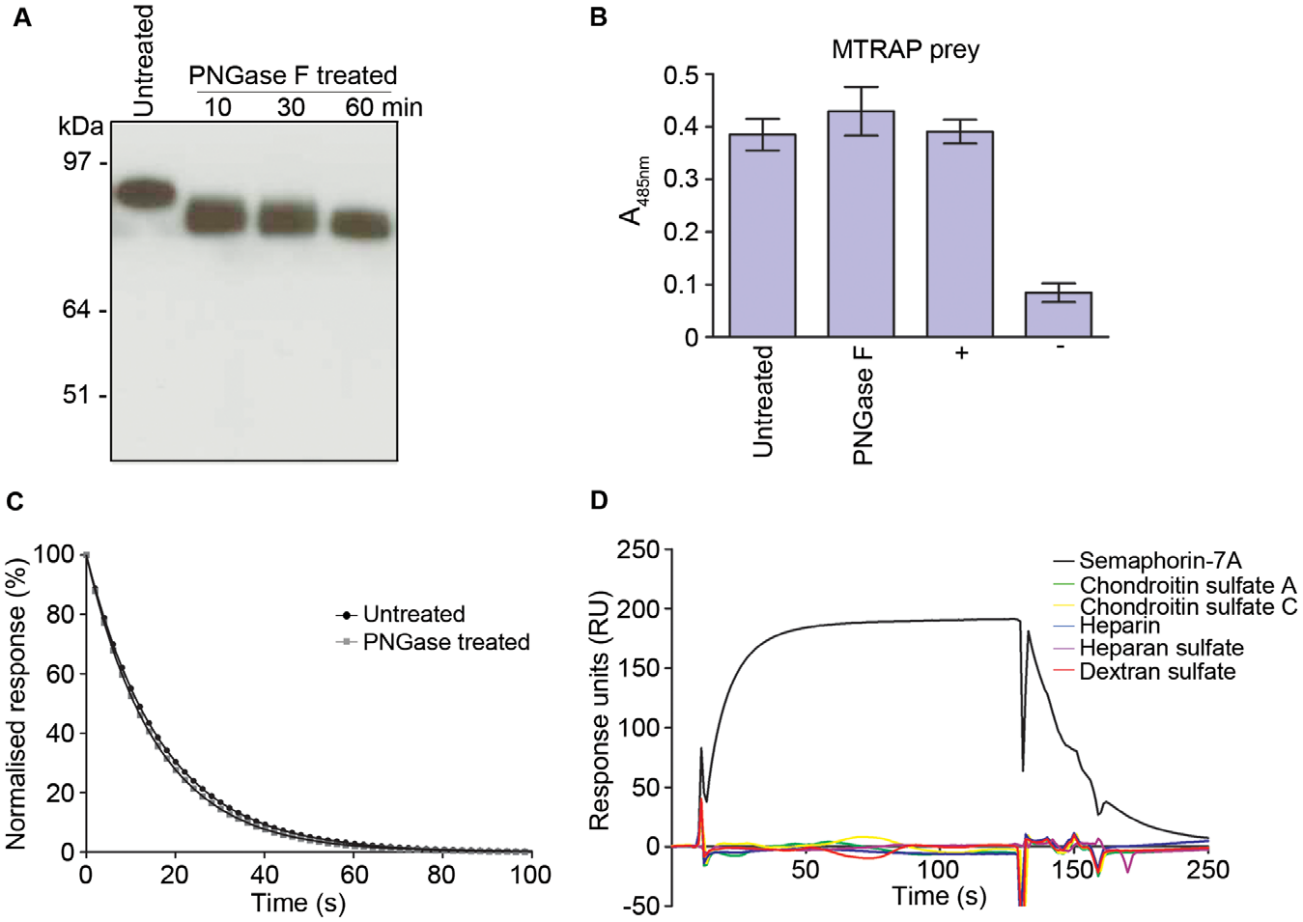
The MTRAP-Semaphorin-7A interaction is not influenced by glycans. (**A**) PNGase F treatment of Semaphorin-7A. Biotinylated Semaphorin-7A was incubated with PNGase F for 10, 30 or 60 minutes. Enzyme-treated and untreated Semaphorin-7A were resolved by SDS-PAGE under reducing conditions and detected by Western blotting using Streptavidin-HRP. (**B**) Binding of MTRAP to PNGase F-treated Semaphorin-7A was indistinguishable from untreated Semaphorin-7A using the AVEXIS assay. MTRAP was used as the prey against Semaphorin-7A baits. (+) = positive control, (−) = negative control. Bar graphs show mean ± SEM, n = 3. (**C**) PNGase F treatment did not quantitatively influence MTRAP binding to Semaphorin-7A using SPR. Three concentrations of purified monomeric MTRAP were injected over flow cells immobilised with PNGase F-treated and untreated Semaphorin-7A. Dissociation rate constants (*k*_d_) were calculated to be 0.063±0.00007 s^−1^ for PNGase F treated, and 0.061±0.00006 s^−1^ for untreated Semaphorin-7A, by fitting a first order dissociation model to the washout phase of the binding curves. Shown are the normalized, averaged values ± SEM, n = 3. (**D**) MTRAP does not interact with sulphated glycoconjugates. Purified monomeric Semaphorin-7A, chondroitin sulphate A, chondroitin sulphate C, dextran sulphate, heparin and heparan sulphate were injected at 1 mg/ml over MTRAP immobilised on a streptavidin-coated sensor chip.

The interaction interface on erythrocyte receptors bound by merozoite surface ligands have been shown, in several cases, to be dependent on the glycosylation state of their erythrocyte receptors [34], [35], [36]. Human Semaphorin-7A contains five predicted N-linked glycosylation sites and so to determine whether MTRAP binding was influenced by glycans, we treated recombinant Semaphorin-7A with PNGase F to remove N-linked glycans (Figure 2 A). PNGase F-treated Semaphorin-7A was indistinguishable from untreated Semaphorin-7A in its ability to bind MTRAP using either the AVEXIS assay (Figure 2 B) or more quantitative SPR (Figure 2 C). These data suggest that the interaction of MTRAP with Semaphorin-7A is not influenced by the presence of glycans on the receptor.

It is known that TRAP can bind sulphated glycoconjugates on hepatocytes [19]. To investigate whether MTRAP was able to bind sulphated glyconjugates, we tested a panel of natural and synthetic glycoconjugates and determined whether they could bind MTRAP using SPR. Chondroitin sulphate A, chondroitin sulphate C, dextran sulphate, heparin and heparan sulphate were each injected at high concentrations over MTRAP immobilised on a sensor chip. No detectable binding for any of the glycoconjugates was observed relative to the Semaphorin-7A positive control (Figure 2 D). We estimate that interactions as weak as 100 µM would have been detected using this approach and conclude that glycoconjugates are unlikely to be major MTRAP ligands.

### Semaphorin-7A and MTRAP form an equimolar complex

**Figure 3:**
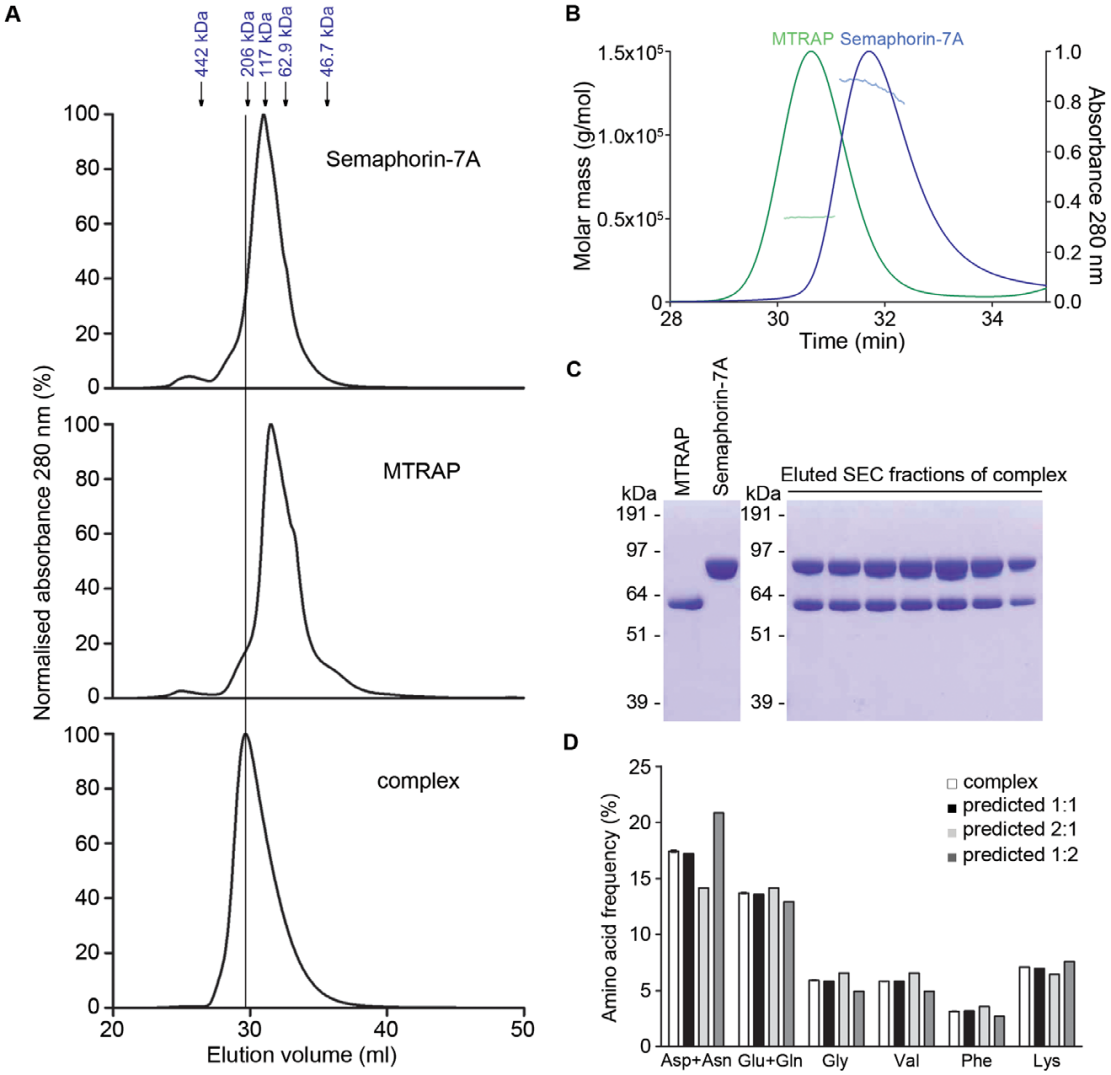
Semaphorin-7A and MTRAP form an equimolar complex. (**A**) Size exclusion chromatography of purified His-tagged Semaphorin-7A and MTRAP, individually and as a complex. Semaphorin-7A (with Cd4-tag; top panel) and MTRAP (without Cd4-tag; middle panel) were analysed by SEC separately and as a complex (bottom panel). (**B**) Multiangle light scattering of purified His-tagged Semaphorin-7A and MTRAP. Semaphorin-7A (with Cd4-tag; blue) and MTRAP (without Cd4-tag; green) were analysed by SEC immediately followed by MALS. Peaks correspond to SEC elution as a function of time with absorbance at 280 nm on the right y-axis, and the horizontal lines indicate molecular mass on the left y-axis. (**C**) SDS-PAGE analysis of the Semaphorin-7A∶MTRAP complex. SEC fractions of the Semaphorin-7a∶MTRAP complex (Figure 3 A) were analysed by SDS-PAGE and visualised by Coomassie staining. The middle fraction, corresponding to the highest point of the peak, was used for amino acid analysis. (**D**) Amino acid analysis of the MTRAP and Semaphorin-7A complex reveals a 1∶1 binding stoichiometry. The fraction of the highest point of the SEC peak of mixed purified MTRAP and Semaphorin-7A was analysed by amino acid analysis. The amino acid composition agrees best with 1∶1 binding. Bars indicate mean ± SEM n = 3.

Structural and biochemical studies have shown that semaphorins exist as homodimers [37], [38], [39]. Size exclusion chromatography (SEC) confirmed that our recombinant soluble monomeric Semaphorin-7A ectodomain eluted in a fraction consistent with it forming a homodimer (Figure 3 A; top panel), as has been shown before [39]. Surprisingly, the ectodomain of MTRAP also eluted at an increased size, perhaps suggesting it also forms a homodimer in solution (Figure 3 A; middle panel). To further investigate these findings, both proteins were subjected to SEC immediately followed by multiangle light scattering (MALS). This analysis demonstrated that while soluble Semaphorin-7A formed quasi-stable homodimers, the soluble MTRAP ectodomain was monomeric (Figure 3 B). The early elution behavior of MTRAP in SEC may therefore be caused by a large hydrodynamic shape possibly due to the protein being highly flexible or adopting a long rod-like shape, as has recently been suggested from atomic force microscopy studies [40]. To determine the stoichiometry of the Semaphorin-7A:MTRAP complex, both purified proteins were mixed at equimolar concentrations and allowed to form a complex prior to separation by SEC. As expected, the complex eluted at a higher mass than each protein individually (Figure 3 A; bottom panel) and both proteins were present in these fractions (Figure 3 C). The unusual behaviour of these proteins by SEC made determining the stoichiometry of the complex by this method difficult and so the fraction corresponding to the peak was subjected to amino acid analysis (Figure 3 D). The amino acid compositions determined experimentally were compared to expected theoretical stoichiometries of 1∶1, 2∶1 and 1∶2 (Semaphorin-7A∶MTRAP). The amino acids that were most characteristic of either Semaphorin-7A or MTRAP indicated that a 1∶1 ratio best fitted the data (Figure 3 D). Calculating the sum of the squared residuals for all amino acids gave values of 1×10^−4^, 14×10^−4^ and 17×10^−4^ for the 1∶1, 2∶1 and 1∶2 models, respectively; again, indicating that the 1∶1 ratio best fitted the data. These results therefore suggest that two MTRAP monomers bind one Semaphorin-7A homodimer.

### MTRAP and Semaphorin-7A interact via their TSR and Sema domains

**Figure 4:**
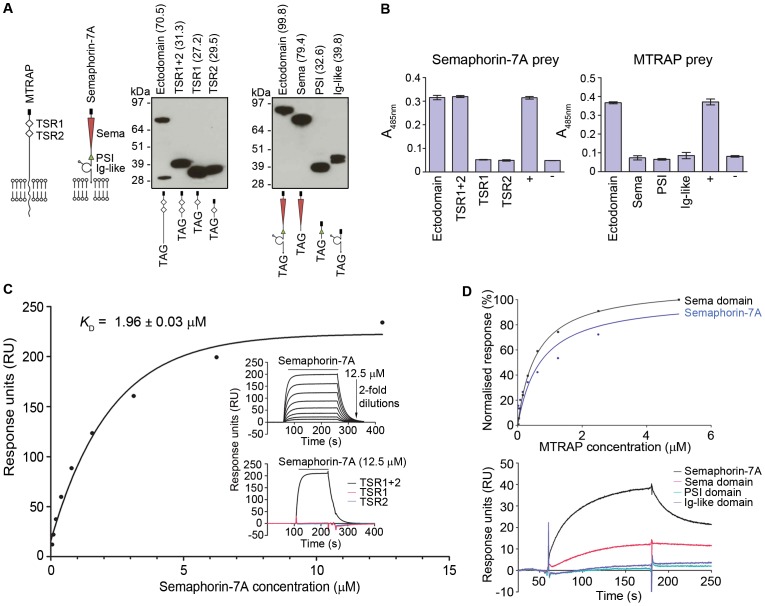
MTRAP and Semaphorin-7A interact via their TSR and Sema domains. (**A**) Expression of individual domains of MTRAP and Semaphorin-7A. Schematics of the MTRAP ligand and Semaphorin-7A receptor as they would appear in the membrane are shown on the left with individual domains labelled plus a signal peptide (black box). The entire ectodomain and subfragments containing the individual domains of MTRAP (left blot) and Semaphorin-7A (right blot) were resolved by SDS-PAGE under reducing conditions and detected by Western blot using Streptavidin-HRP. Predicted molecular weights (kDa) are indicated in brackets; MTRAP has an additional processed band at around 30 kDa corresponding to the size of the Cd4d3+4-tag (TAG = Cd4d3+4-Biotin). (**B**) The two MTRAP TSR domains presented in tandem but not individually, bind Semaphorin-7A using the AVEXIS assay. Biotinylated entire ectodomains and individual domains of MTRAP (left graph) and Semaphorin-7A (right graph) were used as baits in the AVEXIS assay against Semaphorin-7A and MTRAP preys, respectively. The different Semaphorin-7A domains show no binding using this technique. Bar graphs represent mean ± SEM, n = 3. (**C**) MTRAP TSR 1+2 bind Semaphorin-7A with similar kinetics as the entire ectodomain of MTRAP. Serial dilutions of purified Semaphorin-7A were injected over biotinylated TSR1+2 immobilised on a streptavidin-coated sensor chip until equilibrium was reached (upper inset). Reference-subtracted binding data were plotted as a binding curve and an equilibrium dissociation constant calculated as before. A *K*_D_ of 1.96±0.03 µM (mean ± SEM) was calculated from three independent experiments (Table S1). A representative experiment is shown. No binding was observed with TSR1 or TSR2 individually (lower inset). (**D**) The Sema domain binds MTRAP with similar kinetics as the entire ectodomain of Semaphorin-7a. Serial dilutions of purified MTRAP were injected over the biotinylated Semaphorin-7A ectodomain or each individual domains (Sema, PSI and Ig-like) immobilised on a streptavidin-coated sensor chip. Binding was observed with the Sema domain (top graph) with a *K*_D_ of 0.83±0.43 (mean ± SEM), calculated from two independent experiments. No binding was observed with the PSI or Ig domains individually (bottom graph).

The paired TSR domains are the most conserved region of the MTRAP ectodomain across different *Plasmodium* species, and the TSR domain of TRAP has previously been shown to contribute to receptor binding [19]. To investigate whether the TSR domains of MTRAP contain the Semaphorin-7A binding site, a 74 amino acid fragment that contained both predicted TSR domains (TSR1+2), and two additional fragments containing each TSR domain individually, were expressed as biotinylated bait proteins (Figure 4 A). The TSR1+2 MTRAP fragment bound to Semaphorin-7A indistinguishably from the entire MTRAP ectodomain using the AVEXIS assay demonstrating that the Semaphorin-7A binding site was localised to the two TSR domains (Figure 4 B). A quantitative analysis using SPR demonstrated a slightly weaker interaction affinity for TSR1+2 (*K*_D_ = 1.96±0. 03 µM; Figure 4 C; Table S1) compared to the entire MTRAP ectodomain, suggesting that residues outside of the TSR domains make only minor contributions to the binding affinity. Supporting this, purified pentameric TSR1+2 was able to bind erythrocytes (Figure S1), and it has recently been shown that recombinant MTRAP lacking the TSR domains could not [40]. Neither of the two individual TSR domains bound Semaphorin-7A by AVEXIS (Figure 4 B) or SPR (Figure 4 C lower inset) demonstrating that the Semaphorin-7A binding site requires both TSR domains.

Similarly, to localise the MTRAP binding site on Semaphorin-7A, we expressed constructs containing each of the three recognisable domains: Sema, PSI and Ig-like (Figure 4 A). Only the entire ectodomain of Semaphorin-7A bound MTRAP using the AVEXIS assay, irrespective of the bait-prey orientation (Figure 4 B, Figure S2). By SPR, however, detectable binding to MTRAP was observed using the Sema domain alone (Figure 4 D; top graph) with similar binding parameters to the full-length ectodomain (*K*_D_ = 0.83±0.43 µM). No binding was observed with the individual PSI or Ig-like domains (Figure 4 D; bottom graph). Taken together, these data demonstrate Semaphorin-7A and MTRAP directly interact via their Sema and tandem TSR domains respectively.

### Naturally-occurring genetic variants in Semaphorin-7A do not alter MTRAP binding affinity

**Figure 5:**
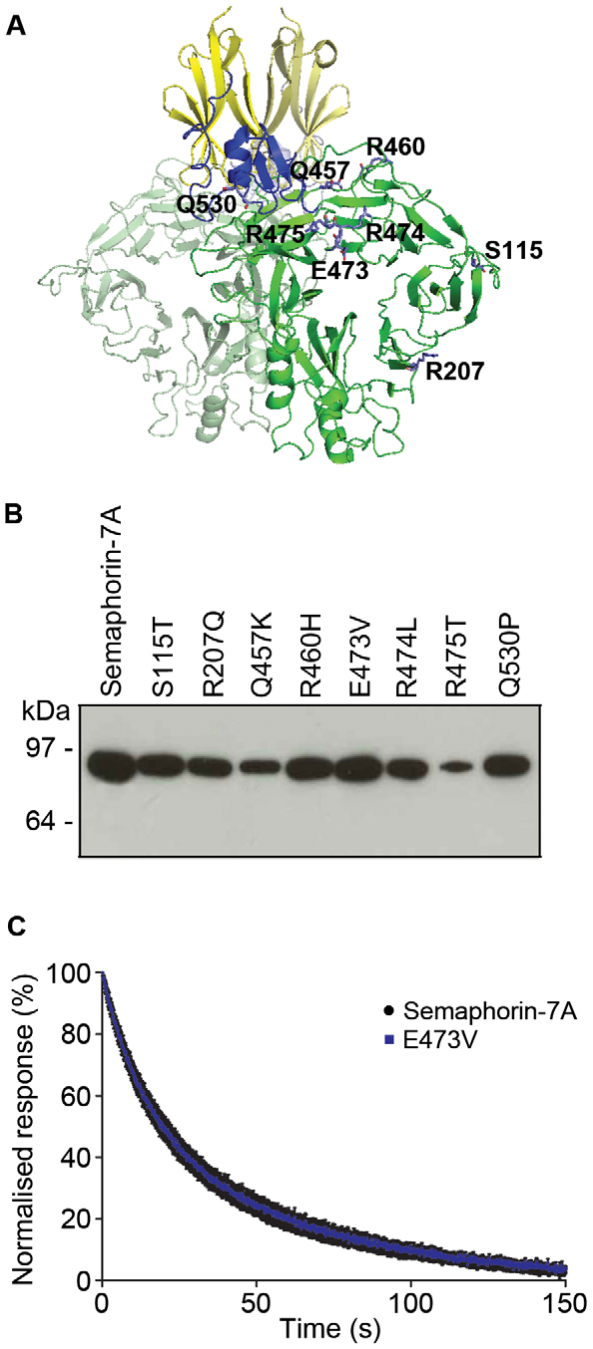
Naturally-occurring variants in Semaphorin-7A do not affect MTRAP binding. (**A**) Representation of the dimeric Semaphorin-7A crystal structure showing the locations of the known naturally-occurring variants (PDB accession code 3NVQ [39]). The Ig-like domain is shown in yellow, the PSI domain in blue and the Sema domain in green. (**B**) Expression of naturally-occurring variants of Semaphorin-7A. All variants were expressed at the expected size as determined by Western blotting using streptavidin-HRP. (**C**) Naturally-occurring variants of Semaphorin-7A do not alter binding to MTRAP as shown by SPR. Serial dilutions of purified MTRAP were injected over Semaphorin-7A and the dissociation rate constant calculated and compared to each of the eight variants; for clarity, only E473V is shown here as a representative example. Dissociation rate constants (*k*_d_) were calculated from two independent experiments and range between 0.050±0.003 s^−1^ and 0.056±0.003 s^−1^ (mean ± SEM) for all variants (Table S3).

Malaria is thought to have been a powerful selective force in human evolutionary history and given the essential role of MTRAP in parasite blood stage culture we asked whether any naturally-occurring polymorphisms in human Semaphorin-7A would influence the binding of MTRAP. Eight variants in the extracellular region of human Semaphorin-7A are known (seven within the Sema domain and one within the PSI domain) and all were individually introduced by site-directed mutagenesis (Figure 5 A; Table S2). All variants were expressed (Figure 5 B) and the dissociation rate constants (*k*_d_) for MTRAP binding were determined using SPR (Figure 5 C, Table S3). No significant differences were observed in the interaction strengths for any of the variants suggesting that at least the known common variants in Semaphorin-7A have not been selected due to differences in the ability to bind *P. falciparum* MTRAP.

### Competing the MTRAP-Semaphorin-7a interaction during erythrocyte invasion

**Figure 6:**
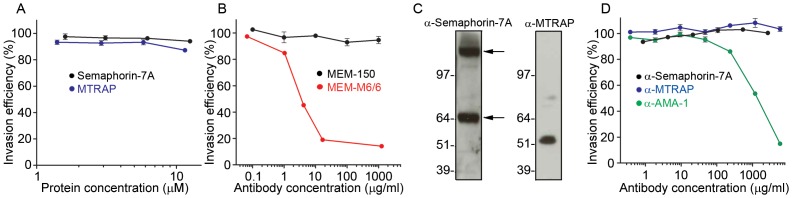
Attempts to block the MTRAP-Semaphorin-7A interaction has no effect on erythrocyte invasion. (**A**) Recombinant Semaphorin-7A and MTRAP have no inhibitory effect on erythrocyte invasion. Purified pentamerised Semaphorin-7A or MTRAP were added to *P. falciparum* erythrocyte invasion assays at concentrations that exceeded the monomeric equilibrium affinity constant by a factor of 10. (**B**) Monoclonal anti-Semaphorin-7A antibody has no effect on erythrocyte invasion. Monoclonal anti-Semaphorin-7A (MEM-150) and an anti-basigin positive control (MEM-M6/6) were added to *P. falciparum* erythrocyte invasion assays in increasing concentrations. MEM-M6/6 showed clear inhibition of invasion, whereas MEM-150 had no observable effect. (**C**) Rabbit polyclonal antibodies against purified monomeric MTRAP and Semaphorin-7A bind native MTRAP and Semaphorin-7A. Erythrocyte ghosts (left blot) and parasite supernatants (right blot) were analysed by Western blot under non-reducing conditions, and detected by incubation with purified polyclonal antibodies, followed by an anti-rabbit-IgG-HRP antibody. The predicted monomer molecular weights of native Semaphorin-7A and MTRAP are 79.3 and 55.6 kDa respectively. The top arrow indicates the dimer and the bottom arrow the monomer of Semaphorin-7A. (**D**) Polyclonal antibodies against MTRAP and Semaphorin-7A do not inhibit erythrocyte invasion. Purified polyclonal antibodies against MTRAP and Semaphorin-7A were added to *P. falciparum* erythrocyte invasion assays but did not affect the efficiency of invasion relative to a positive control (anti-AMA-1).

To examine the role of the MTRAP-Semaphorin-7A interaction in erythrocyte invasion, we attempted to block invasion *in vitro* using purified recombinant proteins and antibodies raised against either the parasite ligand or erythrocyte receptor. Addition of purified highly avid pentamerised Semaphorin-7A or MTRAP in increasing concentrations had no inhibitory effect on erythrocyte invasion (Figure 6 A), even at concentrations 10-fold higher than the measured interaction strength between monomeric proteins (Figure 1 D). Previous studies of the PfRH5-basigin interaction suggest that antibodies more potently block receptor-ligand interactions during erythrocyte invasion, presumably because their interaction affinities are much higher. We therefore tested an anti-Semaphorin-7a monoclonal antibody in *P. falciparum* invasion assays at increasing concentrations. No inhibitory activity was seen even at the highest concentrations, unlike a monoclonal directed against the PfRH5 receptor, basigin, which has a >80% invasion inhibitory effect at 10 µg/ml (Figure 6 B). We also raised rabbit polyclonal antibodies against purified, recombinant, monomeric MTRAP and Semaphorin-7A. Both antibodies were able to detect proteins of the expected size in parasite supernatants (MTRAP) and erythrocyte ghost preparations (Semaphorin-7A) by Western blot (Figure 6 C); we also showed that the anti-MTRAP antibodies were able to block binding of MTRAP to Semaphorin-7A by AVEXIS (Figure S3). When added to invasion assays, however, neither had an inhibitory effect on *P. falciparum* erythrocyte invasion, even at the highest concentration, in contrast to antibodies against AMA-1 (Figure 6 D). Other attempts to block invasion through antibodies targeting MTRAP have yielded similar results [10], [40], suggesting that the MTRAP-Semaphorin-7A interaction is either not accessible to blocking agents in *in vitro* assays, perhaps because it takes place at a late time point during the invasion process, or it is not essential for erythrocyte invasion.

## Discussion

In this study, we have successfully expressed a functional recombinant *P. falciparum* MTRAP protein and shown that it binds erythrocytes. This protein and a library of human erythrocyte receptor ectodomains were used to identify Semaphorin-7A as its erythrocyte receptor using a systematic screening assay (AVEXIS) that is specifically designed to detect low affinity extracellular protein interactions. Importantly, this represents the first example of a host cell surface receptor protein for the TRAP-like family of parasite ligands that provide the crucial link between the target host cell and the parasite's cytoplasmic actin-myosin motor that powers the invasion process in any *Plasmodium* species. Saturation binding behaviours showed that the MTRAP-Semaphorin-7A interaction was specific and, as expected, was of moderately low affinity as is typical of other measured extracellular receptor-ligand interactions [24] and is consistent with low recovery of bound recombinant MTRAP to erythrocytes performed by others [40].

The biochemical characterisation of the interaction suggests that two MTRAP monomers interact via their tandem TSR domains with the Sema domains of a Semaphorin-7A homodimer. This result is supported by the recent finding that a recombinant MTRAP protein lacking the TSR domains was unable to bind erythrocytes [40], whereas a protein containing just the two TSR domains could (Figure S1). This was not unexpected as the TSR domains contribute to binding of other TRAP family proteins to their host cells [5], [19] and this region is conserved across MTRAP orthologues in other *Plasmodium* species [10]. In contrast to the sporozoite TRAP protein, we could find no evidence that MTRAP bound sulphated glycoconjugates despite using a highly sensitive assay. This might be explained by the presence of the sequence “WSPCSVTC” in the TSR domains of TRAP, TLP and the related protein CSP which is believed to be a sulfatide binding motif (Muller et al., 1993) and is absent from MTRAP. Recent work by Uchime *et al.* confirmed that the TSR domains of MTRAP structurally differ from previously studied TSR domains based on its disulphide bonds suggesting a more compact structure, perhaps indicating that both TSR domains function together [40] and explaining the requirement for tandem TSR domains in Semaphorin-7A binding.

Our experiments to define the MTRAP binding site on Semaphorin-7A were complicated by the fact that Semaphorin-7A, like other semaphorins, is known to form a dimer with a large (2860 Å^2^) and primarily hydrophobic contact interface that involves the whole molecule, including the Ig domain [39]. Individually expressing each of the constituent domains to map the MTRAP binding site therefore disrupted this homodimeric structure. MTRAP, however, did interact with a construct containing the Sema domain alone immobilised at sufficient density on a Biacore chip (Figure 4 D) suggesting that the Sema domain contained the MTRAP interaction interface. We also established that the stoichiometry of binding is likely to be two MTRAP monomers binding to a single Semaphorin-7A homodimer. This binding model is also used by the endogenous semaphorin ligands, the plexins, as shown by crystallisation of the complex [39], [41]. It is possible, as for the plexins, that binding of MTRAP to a dimeric receptor triggers local MTRAP clustering which is then necessary for function by bringing the cytoplasmic regions into close proximity.

Attempts to genetically disrupt *MTRAP* in multiple *P. falciparum* strains were unsuccessful suggesting that it is essential for blood stage growth [10]. We therefore attempted to disrupt the MTRAP-Semaphorin-7A interaction; however, neither purified highly-avid pentameric proteins of both MTRAP or Semaphorin-7A nor polyclonal antibodies raised against either MTRAP or Semaphorin-7A showed any discernible effect on erythrocyte invasion *in vitro*. The inability of polyclonal antibodies raised against MTRAP to affect invasion is consistent with findings from other groups and suggests that MTRAP is unlikely to be a component of an effective subunit blood-stage vaccine [10], [40]. MTRAP may therefore have an important receptor-independent role similar to TRAP which is required not only for cellular invasion but also gliding motility [13]. One other possible explanation is that the interaction may occur in close physical proximity to the moving junction - an electron dense thickening formed at the nexus of the erythrocyte and merozoite plasma membranes, which would almost certainly be inaccessible to large soluble proteinaceous blocking reagents. This hypothesis can be supported by the lack of non-synonymous polymorphisms found in the MTRAP ectodomain, suggesting that unlike other merozoite ligands it is not exposed to strong selection pressure by the immune system [10]. In agreement with this, MTRAP does not appear to be a primary target of the adaptive immune system, with low anti-MTRAP reactivity in human sera from malaria endemic regions [40]. This is in clear contrast to the highly polymorphic sporozoite TRAP protein [19]. Similarly, Semaphorin-7A had very few known polymorphisms, all of which did not quantitatively affect its interaction with MTRAP, and to our knowledge, there is no evidence of selective pressure on this receptor in malaria endemic regions. Interestingly, loss of Semaphorin-7A expression on erythrocytes can be acquired, typically with increasing age, and levels of erythrocyte Semaphorin-7A expression have been observed to fluctuate significantly during pregnancy [42]. Our finding that Semaphorin-7A is a receptor for *P. falciparum* MTRAP makes correlating the levels of cell surface Semaphorin-7A with clinical parameters of *P. falciparum* infection an important area for future study.

Our finding that Semaphorin-7A is a receptor for MTRAP provides the first example of a host receptor protein for a member of the TRAP-like family. Semaphorin-7A, similar to MTRAP, is a member of a larger family of cell surface proteins, the semaphorins, that can be subdivided into eight different structural classes [43]. Whether the other members of the TRAP-like family will have identifiable receptors within the broader semaphorin family is a key area for future research.

Whether the essentiality of MTRAP simply lies in its function as a membrane-tethered link to the parasite cytoplasmic myosin-based motor or has additional roles in determining the cellular tropism of invasion is still not clear. However, the identification of an erythrocyte receptor for the extracellular region of MTRAP supports a mechanism whereby TRAP-family ligands directly interact with a protein displayed on the surface of the target cell. This interaction may therefore provide the traction required to couple the activity of the parasite myosin-based motor into a relative cellular movement that is necessary for invasion. We believe that this finding together with the successful demonstration of an experimental approach to identify host receptors for parasite TRAP-like ligands will stimulate further research into the challenging task of identifying receptors for this important class of parasite ligands.

## Materials and Methods

### Ethics statement

Use of erythrocytes and serum from human donors for *P. falciparum* culture was approved by the NHS Cambridgeshire 4 Research Ethics Committee. All subjects provided written informed consent. The use of animals to raise antisera was performed according to UK Home Office governmental regulations and approved by the local Sanger Institute ethical review board.

### Recombinant protein production and purification

A list of the erythrocyte receptor proteins used in this study and the numbering used in Figure 1 C can be found in Supplementary Table 1 in Crosnier et al., 2011. Proteins within the human erythrocyte protein library were produced as bait and prey constructs as previously described [26]. Briefly, for the proteins containing a signal peptide, each expression construct contained the entire extracellular region (including the native signal peptide) flanked by unique NotI and AscI sites to facilitate cloning into a vector containing a C-terminal rat Cd4d3+4-tag and either an enzymatically biotinylatable peptide (baits) or a peptide from the rat cartilage oligomeric matrix protein (COMP) which spontaneously forms pentamers followed by the enzyme beta-lactamase (preys). The ectodomain fragments of the four type II proteins (which lack a signal peptide) were expressed only as monomeric baits and not preys. Baits for the type II proteins were produced by flanking the predicted extracellular regions with NotI and AscI restriction enzymes and cloning them into a vector containing a mouse immunoglobulin kappa light chain signal peptide followed by the biotinylatable tag and Cd4d3+4 at the N-terminus of the insert. Bait proteins were enzymatically biotinylated during expression by cotransfection of a secreted form of the *E.coli* BirA protein biotin ligase [25]. The MTRAP ectodomain bait and prey constructs differed from the erythrocyte receptors in that the low-scoring endogenous signal peptide was replaced by a high-scoring signal peptide from a mouse immunoglobulin kappa light chain and the serines and threonines in the context of potential N-linked glycan sites were systematically mutated to alanine to prevent inappropriate glycosylation. All ectodomains were codon optimised for mammalian expression and chemically synthesized (Geneart AG, Regensburg, Germany). The constituent Sema, PSI and Ig-like domains of human Semaphorin-7A were produced by identifying the domain boundaries using the crystal structure of the Semaphorin-7A extracellular region as a guide [39]. The MTRAP TSR1+2, TSR 1 and TSR 2 domain boundaries were estimated based on the location of conserved cysteine residues identified by protein alignments of TSR repeats. The sequences corresponding to these domains were amplified using primers with flanking NotI and AscI cloning sites for cloning into the appropriate expression vectors. The PSI, Ig and all TSR domains were cloned into the same vectors as *MTRAP* to add an exogenous signal peptide required for protein secretion. Naturally-occurring variants of Semaphorin-7A were found in dbSNP (www.ncbi.nlm.nih.gov/projects/SNP/). Constructs containing these variants were produced by site directed mutagenesis (GeneArt AG). Variants were mapped onto the structure of Semaphorin-7A using PyMOL (www.pymol.org). Monomeric His-tagged proteins were prepared by subcloning the NotI/AscI flanked extracellular regions into a vector containing a C-terminal Cd4d3+4 tag followed by a hexa-His tag [25]. An additional monomeric His-tagged MTRAP lacking the Cd4d3+4-tag was produced by amplifying the *MTRAP* coding region with primers containing NotI and EcoRI sites and inserting into a NotI/EcoRI-digested His-vector using standard cloning procedures. Pentameric His-tagged proteins were similarly made by cloning the inserts into a NotI/EcoRI-digested prey vector where the COMP-beta-lactamase region had been replaced by a COMP-hexa-His tag. All proteins were expressed as secreted proteins by transient transfection of the human HEK293E cell line grown in suspension as described [25], [44].

His-tagged proteins were purified from supernatants from transient transfections on HisTrap HP columns (GE Healthcare) using an ÄKTAxpress (GE Healthcare) according to manufacturer's instructions. Size exclusion chromatography of nickel purified samples was carried out on a Superdex 200 Tricorn 10/600 column (GE Healthcare) in HBS-EP (GE Healthcare).

Amino acid analysis was performed by the PNAC Facility, University of Cambridge, Cambridge, UK.

For PNGase F treatment, biotinylated Semaphorin-7A was incubated with 50 U/µl of PNGase F (NEB) for 10, 30 and 60 min at 37°C for Western blot analysis, and 60 min at 37°C for AVEXIS and Biacore analysis.

The *P. falciparum* AMA-1 ectodomain was produced in a similar way to MTRAP, cloned into the vector containing a C-terminal Cd4d3+4 tag followed by a hexa-His tag, then expressed and purified as described above.

### Interaction screening by AVEXIS

Interaction screening was carried out as previously described [25], [26]. Briefly, both bait and prey protein preparations were normalised to activities that have been previously shown to detect transient interactions (monomeric half-lives less than 0.1 second) with a low false positive rate [25]. Biotinylated baits that had been dialysed against HBS were immobilised in the wells of a streptavidin-coated 96-well microtitre plate (NUNC). Normalised preys were added, incubated for 1 hour at room temperature, washed three times in HBS plus 0.1% Tween- 20, and once in HBS, after which 125 µg/ml of nitrocefin was added and absorbance values measured at 485 nm on a Pherastar plus (BMG laboratories). For the screen, a positive control interaction using rat Cd200 as a bait and rat Cd200R as a prey, and a negative control interaction using rat Cd4d3+4 as a bait and rat Cd200R as a prey, was used (+ and − in Figure 1 C). Where AVEXIS was used for interaction site mapping and PNGase F experiments (Figure 2 B and 4 B), the Cd4d3+4 tag alone was used as a negative control bait, and a biotinylated anti-Cd4 antibody as positive control to capture the Cd4d3+4-tagged prey.

### Erythrocyte binding assays

Erythrocyte binding assays were carried out as described previously [45] but with slight modifications. Briefly, 60 µg of purified proteins were mixed with 50 µl of packed fresh erythrocytes for 2 hours at 4°C. The erythrocytes were separated from supernatant by spinning through 400 µl of ice cold dibutyl phthalate (Sigma) at 12000 *g* for 30 seconds, after which the erythrocyte pellet was washed in ice cold PBS. Proteins bound to the erythrocytes were eluted by incubating with 20 µl of 1.5 M NaCl at room temperature for 45 minutes, and collected after 30 seconds of 12,000 *g* centrifugation. The unbound, wash and eluted material were analysed by Western blotting as described below.

### Erythrocyte ghost preparation

Erythrocytes were pelleted then washed twice in 5 volumes of ice cold 2 mM HEPES, 154 mM NaCl, pH 7.1 and centrifuged for 15 mins at 500 *g* at 4°C. The pellet was transferred into 15 volumes of ice cold 10 mM Tris-HCl 1 mM EDTA pH 7.1 and left on ice for 30 mins. After centrifugation at 20,000 *g* for 15 mins at 4°C, the supernatant was discarded and the pellet gently resuspended whilst leaving behind the denser dark pellet of unlysed cells. The pellet was centrifuged at 20,000 *g* for 15 mins at 4°C then washed in 2 mM HEPES, 154 mM NaCl, pH 7.1 four times. The washed ghosts were resuspended in 10 mM Tris-HCl pH 7.1 and centrifuged at 20 000 *g* for 15 mins at 4°C, after which, the pellet was resuspended in 1 volume of 10 mM Tris-HCl pH 7.1, then stored at −20°C.

### Parasite supernatant preparation

To make culture supernatants, synchronised schizonts were purified by centrifugation onto an 80% Percoll cushion, collected at the cushion interface, placed back into in vitro culture at 2.5×10^7^ parasites/ml in the absence of additional erythrocytes and allowed to rupture overnight. Cells were removed by centrifugation and supernatants stored at −80°C.

### Antibodies

To raise polyclonal antisera against Semaphorin-7A, MTRAP, and AMA-1, purified proteins were injected into rabbits (Cambridge Research Biochemicals, Billingham, UK). The sera were purified on Hi-Trap Protein G HP columns (GE Healthcare), and the mouse anti-human Semaphorin-7A, MEM-150 monoclonal antibody [46] was purified from mouse ascites on a HiTrap IgM Purification HP column (GE Healthcare), using an ÄKTA Xpress (GE Healthcare) according to the manufacturer's instructions.

### Western blotting

Proteins were resolved by SDS-PAGE using NuPAGE 4–12% Bis Tris precast gels (Invitrogen). Where reducing conditions were required NuPAGE reducing agent and anti-oxidant (Invitrogen) were added to the sample and the running buffer, respectively. Proteins were blotted onto PVDF membranes (Amersham) and blocked in 2% BSA. Membranes were incubated with either peroxidase-conjugated streptavidin (Jackson Immuno Research) or anti-C-term His-HRP antibody (Invitrogen) as appropriate, and proteins detected using SuperSignal West Pico Chemiluminescent substrate (Thermo Scientific). When using rabbit-anti-Semaphorin-7A or anti-MTRAP, an anti-rabbit-IgG-HRP (Invitrogen) secondary antibody was used.

### Surface plasmon resonance

Surface plasmon resonance studies were performed using a Biacore T100 instrument (GE Healthcare). Biotinylated bait proteins were captured on a streptavidin-coated sensor chip (GE Healthcare). Approximately 150 RU of the negative control bait (biotinylated rat Cd4d3+4) were immobilised in the flow cell used as a reference and approximate molar equivalents of the query protein immobilized in other flow cells. Purified analyte proteins were separated by size exclusion chromatography on a Superdex 200 Tricorn 10/600 column (GE Healthcare) in HBS-EP (GE Healthcare) just prior to use in SPR experiments to remove any protein aggregates that might influence kinetic measurements. Increasing concentrations of purified proteins were injected at 100 µl/min to determine kinetic parameters, or at 20 µl/min for equilibrium measurements. The surface was regenerated with a pulse of 2 M NaCl at the end of each cycle. Duplicate injections of the same concentration in each experiment were super imposable demonstrating no loss of activity after regenerating the surface. Both kinetic and equilibrium binding data were analysed in the manufacturer's Biacore T100 evaluation software version 1.1.1 (GE Healthcare). Equilibrium binding measurements were taken once equilibrium had been reached using reference-subtracted sensorgrams. Both the kinetic and equilibrium binding were replicated using independent protein preparations of both ligand and analyte proteins. All experiments were performed at 37°C in HBS-EP.

Sulfated-glycoconjugates were obtained from Sigma and resuspended in 1× HBS-EP (Biacore, GE Healthcare) and used for surface plasmon resonance studies at 1 mg/ml.

### Multi-angle light scattering measurements (MALS)

Size exclusion chromatography was performed on a Superdex200 10/30 column (GE Healthcare) equilibrated in 50 mM Tris.HCl, pH 7.5, 150 mM NaCl at 0.4 ml/min. The column was followed in-line by a Dawn Heleos-II light scattering detector (Wyatt Technologies) and an Optilab-Rex refractive index monitor (Wyatt Technologies). Molecular mass calculations were performed using ASTRA 5.3.4.14 (Wyatt Technologies) assuming a dn/dc value of 0.186 ml/g.

### *P. falciparum* culture and invasion assays

The 3D7 *P. falciparum* parasite strain was cultured in human O+ erythrocytes at 5% hematocrit in complete medium (RPMI-1640 containing 10% human serum), under an atmosphere of 1% O_2_, 3% CO_2_, and 96% N_2_.

Invasion assays were carried out in round-bottom 96-well plates, with a culture volume of 100 µL per well at a hematocrit of 2%. Parasites were synchronized at early stages with 5% (w/v) D-sorbitol (Sigma), trophozoite stage parasites were mixed with the specified protein blocking reagent, and then incubated in the plates for 24 hours at 37°C inside a static incubator culture chamber (VWR), gassed with 1% O_2_, 3% CO_2_, and 96% N_2_. At the end of the incubation period, erythrocytes were harvested and parasitized erythrocytes were stained with 2 µM Hoechst 33342 (Invitrogen), as described previously [47]. Purified MEM-150, rabbit polyclonal antibodies and pentamerised MTRAP and Semaphorin-7A ectodomains were dialysed into RPMI (GIBCO) prior to use. A monoclonal antibody targeting basigin (MEM-M6/6, Abcam, Cambridge, UK) was purchased and dialysed into RPMI before addition into invasion assays.

Hoechst 33342 (Invitrogen) stained samples were excited with a 355 nm UV laser (20 mW) on a BD LSRII flow cytometer (BD Biosciences) and detected with a 450/50 filter. BD FACS Diva (BD Biosciences) was used to collect 100,000 events for each sample. FSC and SSC voltages of 423 and 198, respectively, and a threshold of 2,000 on FSC were applied to gate the erythrocyte population. The data collected were further analyzed with FlowJo (Tree Star). All experiments were carried out in triplicate. GraphPad Prism (GraphPad Software) was used to plot the generated parasitemia data.

## Supporting Information

Figure S1 **Purified pentameric TSR1+2 domains bind erythrocytes.** Unbound, wash and eluted material was resolved under reducing conditions by SDS-PAGE and detected by Western blotting using an anti-His antibody. The pentamers split into monomers upon reduction, with a predicted monomer molecular weight of 16.8 kDa.
(PDF)

Figure S2 **The entire ectodomain of Semaphorin-7A binds MTRAP but not the constituent Sema, PSI or Ig-like domains tested individually by AVEXIS.** The entire ectodomain of Semaphorin-7A and each constituent domain (Sema, PSI and Ig-like) were produced as pentameric preys and tested for binding using the AVEXIS assay with baits comprising either the entire MTRAP ectodomain or the two TSR domains. Binding was observed with the entire Semaphorin-7A ectodomain but not with any of the three domains presented individually. The Cd4d3+4-tag was used as a negative control and an anti-Cd4d3+4 antibody (OX68) as a positive control. Bar chart represent means ± SEM, n = 3.
(PDF)

Figure S3 **Anti-MTRAP antibodies are able to block MTRAP binding to Semaphorin-7A.** MTRAP beta-lactamase-tagged “prey” protein was incubated with serial dilutions of anti-MTRAP antisera, before being tested for binding to a Semaphorin-7A “bait” captured on a microtitre plate by the AVEXIS assay. Positive binding is indicated by absorbance at 485 nm by the hydrolytic products of a colorimetric beta-lactamase substrate, nitrocefin. Anti-MTRAP antibodies (blue circles) exhibited a dose-dependent inhibition of binding relative to a control antibody (red squares). Data points represent means ± SD, n = 3.
(PDF)

Table S1 **Summary of the biophysical binding data for the MTRAP-Semaphorin-7A interaction.** The equilibrium and kinetic measurements were calculated from surface plasmon resonance studies using serial dilutions of Semaphorin-7A as the analyte and MTRAP or TSR1+2 as the immobilised ligands. The experiment was performed three times using independently produced protein samples. The parameters from each experiment are derived by fitting a steady state affinity (equilibrium) and a dissociation (kinetic) model to a family of binding curves produced from dilution series of the analyte proteins.
(PDF)

Table S2 **Non-synonymous single nucleotide polymorphisms (SNPs) located within the predicted extracellular regions of Semaphorin-7A.** Eight non-synonymous polymorphisms have been identified in human Semaphorin-7A, seven of which are located within the Sema domain and one within the PSI domain. Population frequency data exist for only one of these SNPs: rs16968733 which is found in Africa at a frequency of 0.029.
(PDF)

Table S3 **Summary of the biophysical binding data for MTRAP binding Semaphorin-7A and its naturally-occurring sequence variants.** The kinetic measurements were calculated from surface plasmon resonance studies using serial dilutions of MTRAP as analytes and Semaphorin-7a and its variants as the immobilised ligand. The experiment was performed twice using independently produced protein samples. The parameters from each experiment are derived by fitting a dissociation model to a family of binding curves produced from a dilution series of the MTRAP protein.
(PDF)
