# Supplementary figures and images for: Semaphorin-7A Is an Erythrocyte Receptor for P. falciparum Merozoite-Specific TRAP Homolog, MTRAP
Source: PLoS Pathog. 2012 Nov 15;8(11):e1003031. doi: 10.1371/journal.ppat.1003031 (PMC3499583; doi:10.1371/journal.ppat.1003031)

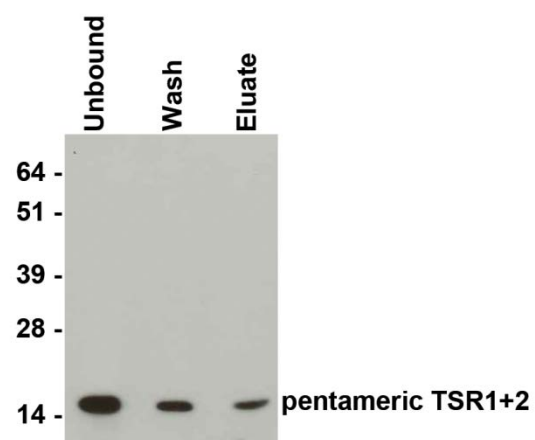

**Figure S1**

Supplement: Figure S1 — Purified pentameric TSR1+2 domains bind erythrocytes. Unbound, wash and eluted material was resolved under reducing conditions by SDS-PAGE and detected by Western blotting using an anti-His antibody. The pentamers split into monomers upon reduction, with a predicted monomer molecular weight of 16.8 kDa. (PDF) [file ppat.1003031.s001.pdf]

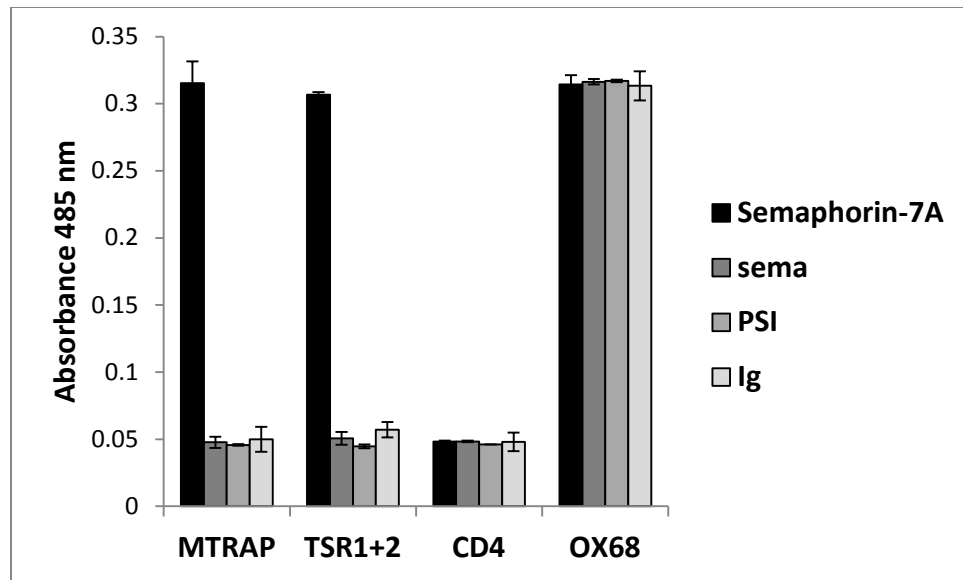

**Figure S2**

Supplement: Figure S2 — The entire ectodomain of Semaphorin-7A binds MTRAP but not the constituent Sema, PSI or Ig-like domains tested individually by AVEXIS. The entire ectodomain of Semaphorin-7A and each constituent domain (Sema, PSI and Ig-like) were produced as pentameric preys and tested for binding using the AVEXIS assay with baits comprising either the entire MTRAP ectodomain or the two TSR domains. Binding was observed with the entire Semaphorin-7A ectodomain but not with any of the three domains presented individually. The Cd4d3+4-tag was used as a negative control and an anti-Cd4d3+4 antibody (OX68) as a positive control. Bar chart represent means ± SEM, n = 3. (PDF) [file ppat.1003031.s002.pdf]

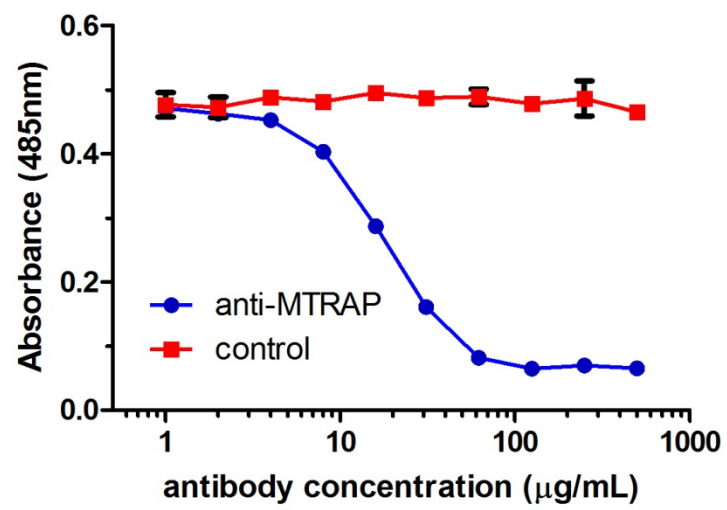

Figure S3

Supplement: Figure S3 — Anti-MTRAP antibodies are able to block MTRAP binding to Semaphorin-7A. MTRAP beta-lactamase-tagged “prey” protein was incubated with serial dilutions of anti-MTRAP antisera, before being tested for binding to a Semaphorin-7A “bait” captured on a microtitre plate by the AVEXIS assay. Positive binding is indicated by absorbance at 485 nm by the hydrolytic products of a colorimetric beta-lactamase substrate, nitrocefin. Anti-MTRAP antibodies (blue circles) exhibited a dose-dependent inhibition of binding relative to a control antibody (red squares). Data points represent means ± SD, n = 3. (PDF) [file ppat.1003031.s003.pdf]
