# Supplementary material for: Semaphorin-7A Is an Erythrocyte Receptor for P. falciparum Merozoite-Specific TRAP Homolog, MTRAP
Source: PLoS Pathog. 2012 Nov 15;8(11):e1003031. doi: 10.1371/journal.ppat.1003031 (PMC3499583; doi:10.1371/journal.ppat.1003031)
Supplement: Table S1 — Summary of the biophysical binding data for the MTRAP-Semaphorin-7A interaction. The equilibrium and kinetic measurements were calculated from surface plasmon resonance studies using serial dilutions of Semaphorin-7A as the analyte and MTRAP or TSR1+2 as the immobilised ligands. The experiment was performed three times using independently produced protein samples. The parameters from each experiment are derived by fitting a steady state affinity (equilibrium) and a dissociation (kinetic) model to a family of binding curves produced from dilution series of the analyte proteins. (PDF) [file ppat.1003031.s004.pdf]

			Equilibrium analysis				Kinetic analysis				
**Experiment #**	**Ligand**	**Analyte**	**K_D (μM)**	**Fit error**	**Mean (μM)**	**SEM**	**k_d (s^{-1})**	**Fit error**	**Mean (s^{-1})**	**SEM**	**$t_{1/2}$ (s)**
1	MTRAP	Semaphorin-7A	1.07	0.95			0.05900	0.00009			
2	MTRAP	Semaphorin-7A	0.85	0.08	1.18	0.40	0.06091	0.00002	0.060	0.001	11.60
3	MTRAP	Semaphorin-7A	1.62	0.18			0.05933	0.00009			
1	TSR1+2	Semaphorin-7A	1.93	0.23			0.06207	0.00001			
2	TSR1+2	Semaphorin-7A	2.00	0.23	1.96	0.03	0.05023	0.00001	0.054	0.007	12.84
3	TSR1+2	Semaphorin-7A	1.94	0.21			0.05008	0.00001			

Table S1
