# Supplementary material for: Semaphorin-7A Is an Erythrocyte Receptor for P. falciparum Merozoite-Specific TRAP Homolog, MTRAP
Source: PLoS Pathog. 2012 Nov 15;8(11):e1003031. doi: 10.1371/journal.ppat.1003031 (PMC3499583; doi:10.1371/journal.ppat.1003031)
Supplement: Table S2 — Non-synonymous single nucleotide polymorphisms (SNPs) located within the predicted extracellular regions of Semaphorin-7A. Eight non-synonymous polymorphisms have been identified in human Semaphorin-7A, seven of which are located within the Sema domain and one within the PSI domain. Population frequency data exist for only one of these SNPs: rs16968733 which is found in Africa at a frequency of 0.029. (PDF) [file ppat.1003031.s005.pdf]

SNP number	Nucleotide variant	Amino acid polymorphism	Location of polymorphism
rs16968733	T>A	S115T	Sema
rs55637216	G>A	R207Q	Sema
rs55757167	C>A	Q457K	Sema
rs56204206	G>A	R460H	Sema
rs56064164	A>T	E473V	Sema
rs56100085	G>T	R474L	Sema
rs56384187	G>C	R475T	Sema
rs55696764	A>C	Q530P	PSI

Table S2
