# Supplementary material for: Semaphorin-7A Is an Erythrocyte Receptor for P. falciparum Merozoite-Specific TRAP Homolog, MTRAP
Source: PLoS Pathog. 2012 Nov 15;8(11):e1003031. doi: 10.1371/journal.ppat.1003031 (PMC3499583; doi:10.1371/journal.ppat.1003031)
Supplement: Table S3 — Summary of the biophysical binding data for MTRAP binding Semaphorin-7A and its naturally-occurring sequence variants. The kinetic measurements were calculated from surface plasmon resonance studies using serial dilutions of MTRAP as analytes and Semaphorin-7a and its variants as the immobilised ligand. The experiment was performed twice using independently produced protein samples. The parameters from each experiment are derived by fitting a dissociation model to a family of binding curves produced from a dilution series of the MTRAP protein. (PDF) [file ppat.1003031.s006.pdf]

Experiment #	Ligand	Analyte	k_d (s^{-1})	Fit error	Mean (s^{-1})	SEM	$t_{1/2}$ (s)
1	Semaphorin-7A	MTRAP	0.058	0.0003	0.056	0.003	12.38
2	Semaphorin-7A	MTRAP	0.054	0.0003			
1	S115T	MTRAP	0.053	0.0005	0.050	0.004	13.86
2	S115T	MTRAP	0.047	0.0009			
1	R207Q	MTRAP	0.054	0.0005	0.050	0.006	14.00
2	R207Q	MTRAP	0.045	0.0007			
1	Q457K	MTRAP	0.053	0.0006	0.056	0.004	12.49
2	Q457K	MTRAP	0.058	0.005			
1	R460H	MTRAP	0.049	0.0007	0.050	0.001	13.86
2	R460H	MTRAP	0.051	0.0003			
1	E473V	MTRAP	0.056	0.0007	0.055	0.001	12.60
2	E473V	MTRAP	0.054	0.0004			
1	R474L	MTRAP	0.046	0.0006	0.052	0.008	13.46
2	R474L	MTRAP	0.057	0.0003			
1	R475T	MTRAP	0.059	0.0014	0.056	0.004	12.38
2	R475T	MTRAP	0.053	0.0004			
1	Q530P	MTRAP	0.05	0.0005	0.053	0.004	13.20
2	Q530P	MTRAP	0.055	0.0002			

Table S3
